# Somatic Symptom Disorder Is Associated with Cough Hypersensitivity and Poor Response to Anti-Reflux Therapy in Patients with Gastroesophageal Reflux-Induced Chronic Cough

**DOI:** 10.3390/jcm15103618

**Published:** 2026-05-08

**Authors:** Yaxing Zhou, Tongyangzi Zhang, Jashin In, Shengyuan Wang, Jiaying Yuan, Bingxian Sha, Haodong Bai, Heng Wu, Li Yu, Xianghuai Xu

**Affiliations:** 1 Department of Pulmonary and Critical Care Medicine, Tongji Hospital, School of Medicine, Tongji University, Shanghai 200065, China; 2 Department of Psychosomatic Medicine, Tongji Hospital, School of Medicine, Tongji University, Shanghai 200065, China

**Keywords:** chronic cough, gastroesophageal reflux-induced chronic cough, somatic symptom disorder, psychosomatic comorbidity

## Abstract

**Background/Objectives:** Gastroesophageal reflux-induced chronic cough (GERC) is a common cause of chronic cough, and a substantial proportion of patients show an inadequate response to standard anti-reflux therapy. Psychological comorbidities, including somatic symptom disorder (SSD), have been increasingly recognized in this population. However, the prevalence and clinical characteristics of SSD in patients with GERC remain unclear. This study aimed to determine the prevalence of SSD in patients with GERC and to characterize the associated clinical and psychological features. **Methods**: In this prospective observational study, consecutive patients with GERC diagnosed at Tongji Hospital, Shanghai, China, between January 2024 and June 2025 were consecutively enrolled. SSD was assessed using a structured diagnostic interview based on DSM-5 criteria. Patients were categorized into SSD+ and SSD− groups. Clinical and psychological characteristics were compared between groups, and binary logistic regression was performed to identify factors associated with SSD. **Results**: A total of 215 patients with GERC were included, of whom 22.3% (48/215) were diagnosed with SSD. Compared with the SSD− group, patients with SSD had higher healthcare utilization, poorer response to standard anti-reflux therapy, increased cough sensitivity, poorer cough-related quality of life, and greater psychological distress. Multivariate logistic regression identified increased cough sensitivity and higher anxiety and depression scores as independent factors associated with SSD. **Conclusions**: SSD is relatively common in patients with GERC and is associated with cough hypersensitivity, poorer response to anti-reflux therapy, and greater psychological burden. These findings suggest that SSD may represent a clinically relevant phenotype of GERC, and patients with coexisting SSD may require more comprehensive psychosomatic assessment and multidisciplinary management.

## 1. Introduction

Cough is one of the most common symptoms in respiratory diseases, and chronic cough accounts for more than one-third of visits to respiratory clinics [1,2]. Gastroesophageal reflux-induced chronic cough (GERC) is defined as gastric acid and other gastric content reflux into the esophagus and trigger cough as the predominant or sole manifestation, representing a distinct subtype of gastroesophageal reflux disease (GERD) [3,4]. GERC is a common cause of chronic cough, accounting for approximately 10–40% of cases [5]. Its pathogenesis is multifactorial, encompassing the “reflux theory,” in which refluxate directly stimulates the airway mucosa, and the “reflex theory,” whereby reflux induces cough via vagal reflex pathways [3,6]. Increasing evidence suggests that patients with chronic diseases, including chronic cough, often experience a substantial burden of psychological or psychiatric comorbidities, which may further influence disease onset and progression [2,7].

Previous research has primarily focused on emotional disorders such as anxiety and depression, which are highly prevalent in patients with GERD [8,9,10]. In contrast, somatic symptom disorder (SSD) differs fundamentally from anxiety and depression; its defining feature is an excessive preoccupation with and emotional response to physical symptoms. According to the Diagnostic and Statistical Manual of Mental Disorders, Fifth Edition (DSM-5), SSD is characterized by one or more somatic symptoms that cause significant disruption to daily life, accompanied by disproportionate thoughts, feelings, or behaviors related to these symptoms or overall health [11]. Importantly, the diagnosis of SSD no longer depends on the presence or absence of an identifiable organic disease but instead emphasizes the patient’s psychological response to symptoms. This implies that SSD may coexist with organic diseases and influence symptom perception and treatment response even when a clear physiological cause such as reflux is present. SSD is common in individuals with chronic diseases and is closely associated with other psychiatric conditions, including anxiety and depression [12,13,14,15,16].

A previous study conducted in general outpatient clinics in China reported an SSD prevalence of 33.8%, highlighting its high prevalence in clinical populations and the need for greater attention [17]. However, data on the prevalence of SSD in GERD and its related conditions—particularly in patients with GERC—remain limited. Evidence regarding its associated factors and potential impact on treatment response is also lacking. Clarifying the epidemiological characteristics and associated factors of SSD in GERC is essential for improving disease management and advancing integrated biopsychosocial care.

To address this gap, the present study aimed to determine the prevalence of SSD in patients with GERC and to characterize its associated clinical and psychological features, with the goal of informing more comprehensive and individualized management strategies for GERC.

## 2. Materials and Methods

### 2.1. Study Design and Participant

**Figure 1:**
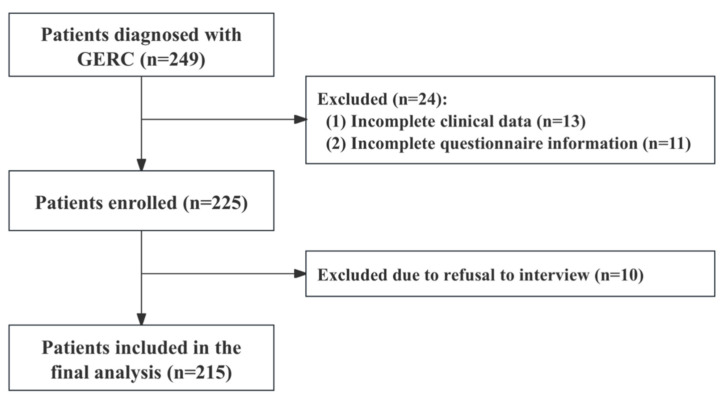
Flowchart of patient selection and inclusion.

This prospective observational study was conducted at the Department of Respiratory and Critical Care Medicine of Tongji Hospital, Shanghai, from January 2024 to June 2025. The study protocol was approved by the Ethics Committee of Tongji Hospital (Approval No. K-2023-027) and registered in the Chinese Clinical Trial Registry (Registration No. ChiCTR2400079808). A total of 215 patients with complete clinical data and GERC as the sole etiology were enrolled. The patient selection process is illustrated in Figure 1. General clinical data was collected, including age, sex, body mass index (BMI), pulmonary function, disease duration, healthcare utilization (number of medical visits), and response to standard anti-reflux therapy. All patients underwent comprehensive cough-related assessments, including the capsaicin cough sensitivity testing, Cough Symptom Score (CSS), Hull Airway Reflux Questionnaire (HARQ), Gastroesophageal Reflux Disease Questionnaire (GerdQ), and Leicester Cough Questionnaire (LCQ). Psychological evaluations were also performed, including the Patient Health Questionnaire-15 (PHQ-15), Somatic Symptom Disorder-12 (SSD-12), Patient Health Questionnaire-9 (PHQ-9), and Generalized Anxiety Disorder-7 (GAD-7). Additionally, all participants underwent multichannel intraluminal impedance–pH (MII–pH) monitoring to assess the characteristics and severity of gastroesophageal reflux.

The inclusion criteria were as follows: (1) age 18–80 years; (2) fulfillment of the diagnostic criteria for GERC as outlined in the *Chinese Guidelines for the Diagnosis and Treatment of Cough (2021)*; (3) completion of cough-related and psychological assessments at the initial visit; and (4) provision of written informed consent.

The exclusion criteria were as follows: (1) language impairment or limited reading comprehension; (2) acute exacerbation of respiratory disease; (3) incomplete clinical data; and (4) failure to complete further psychological evaluation or diagnostic procedures.

#### 2.1.1. Definition of GERC

The diagnosis of GERC was established according to the *Chinese Guidelines for the Diagnosis and Treatment of Cough (2021)* [18]. The diagnostic criteria were as follows:

(1) chronic cough, typically occurring during the daytime, with nocturnal cough present in a minority of patients;
(2) an abnormal acid exposure time (AET) > 6.0% and/or a symptom association probability (SAP) ≥ 9 5% on esophageal reflux monitoring;
(3) significant improvement or resolution of cough following stepwise anti-reflux therapy.

Patients were classified with acid GERC if they met the diagnostic criteria (1) and (3), and had AET > 6.0% and/or acid SAP ≥ 95%. Non-acid GERC was defined in patients who met criteria (1) and (3), with AET ≤ 6.0% and non-acid SAP ≥ 95% [3,19,20].

The diagnostic criteria for refractory GERC were based on previous recommendations [21]. These included:

(1) chronic cough with or without typical reflux symptoms;
(2) pathological reflux (acid or non-acid reflux) confirmed by MII-pH monitoring;
(3) poor response to 8 weeks of standard anti-reflux therapy (e.g., omeprazole 20 mg twice daily), but a good response to intensified anti-reflux treatment, including high-dose proton pump inhibitors (PPIs) or the addition of neuromodulators.

#### 2.1.2. Stepwise Anti-Reflux Treatment

All patients with GERC underwent a standardized stepwise anti-reflux treatment protocol, as outlined below [22]:

(1) initial standard-dose PPI therapy (e.g., omeprazole 20 mg twice daily for 8 weeks);
(2) for patients with inadequate response, intensified therapy with double-dose PPI (e.g., omeprazole 40 mg twice daily) combined with prokinetic agents for 8 weeks;
(3) if symptoms persisted, an H2 receptor antagonist (e.g., ranitidine) was added for 8 weeks;
(4) for persistent non-responders, treatment was escalation to combined anti-reflux and neuromodulator therapy (e.g., baclofen).

If cough symptoms showed improvement at any stage, the corresponding treatment regimen was continued until complete resolution of symptoms; otherwise, patients proceeded to the subsequent step after completing the current treatment phase.

#### 2.1.3. Definition of the SSD

This study established a multidisciplinary team (MDT) clinic for chronic cough, jointly staffed by respiratory physicians and psychosomatic specialists and conducted on a weekly basis. All patients with GERC underwent initial psychological screening at their first respiratory consultation. Patients with abnormal screening results (SSD-12 ≥ 16, PHQ-9 ≥ 5, or GAD-7 ≥ 5) were referred to the MDT clinic for further integrated psychosomatic evaluation.

At the MDT clinic, psychiatric assessment was performed using the Structured Clinical Interview for DSM-5 (SCID) adapted for this study (see Supplementary Material S2). All interviews were conducted by psychiatrists trained in SCID procedures under the supervision of senior psychosomatic specialists.

The diagnosis of somatic symptom disorder (SSD) was established strictly according to DSM-5 criteria and was entirely based on the SCID interview [11]. Specifically, the diagnosis required: (A) one or more distressing somatic symptoms resulting in significant disruption of daily life; (B) excessive thoughts, feelings, or behaviors related to the somatic symptoms or associated health concerns, as manifested by at least one of the following: (1) disproportionate and persistent thoughts about the seriousness of one’s symptoms; (2) persistently high level of anxiety about health or symptoms; (3) excessive time and energy devoted to these symptoms or health concerns; and (C) persistence of the symptomatic state for more than 6 months. All three criteria (A–C) were required for the diagnosis.

Based on the SCID results, patients were classified into SSD (SSD+) and non-SSD (SSD−) groups. The PHQ-15 and SSD-12 were used to assess somatic symptom burden and psychological responses corresponding to DSM-5 criteria A and B, respectively. These instruments were used for quantitative assessment and screening purposes only and were not used as diagnostic criteria. The severity of somatic symptom burden was further categorized according to the PHQ-15 total score (≤9 mild, 10–14 moderate, ≥15 severe).

#### 2.1.4. MII-pH Monitoring Procedure

All participants undergoing MII–pH monitoring discontinued proton pump inhibitors (PPIs) for at least two weeks prior to the examination. The position of the lower esophageal sphincter (LES) was first determined using esophageal manometry (Solar GI, Medical Measurement Systems B.V., Enschede, the Netherlands). A combined MII–pH catheter (2.1 mm diameter; K6011-E10632, Unisensor, Attikon, Switzerland) containing six impedance sensors and an antimony pH electrode (819100, Medical Measurement Systems B.V., Enschede, the Netherlands) was then inserted transnasally. The impedance sensors were positioned at 3, 5, 7, 9, 15, and 17 cm above the LES, and the pH electrode was positioned approximately 5 cm above the LES.

The catheter was connected to a portable data logger (Ohmega, Medical Measurement Systems B.V.) for 24 h ambulatory monitoring. During the recording period, patients were instructed to maintain their normal daily activities and diet, and were instructed to document cough episodes, meal times, and body-position changes on a diary card, and to mark events using the logger’s event button.

Data from all channels were analyzed using dedicated software (MMS database, v8.7). AET was defined as the percentage of monitoring time with esophageal pH < 4. The esophageal DeMeester score was calculated to further assess acid exposure. The temporal relationship between reflux events and cough was evaluated using the symptom index (SI) and SAP. SI represented the percentage of cough episodes preceded by reflux within 2 min, while SAP quantified the statistical probability that this association was not due to chance (significant if ≥95%).

#### 2.1.5. Cough and Psychosomatic-Related Assessment

The CSS was used to assess daytime and nighttime cough severity on a six-point scale (0–5), with 0 indicating no cough and 5 representing the most severe cough [23].

The HARQ, developed by Morice et al., is used to evaluate airway reflux and cough hypersensitivity in patients with chronic cough, with total scores ranging from 0 to 70; higher scores indicate greater cough sensitivity [24]. The Chinese version was translated and validated by our research team and demonstrated good reliability and validity [25].

The GerdQ was used to assess the frequency of typical reflux symptoms (e.g., heartburn, regurgitation) and atypical symptoms over the past 7 days, with total scores ranging from 0 to 18. Higher scores indicate a greater likelihood of GERD [26].

The LCQ, developed by Birring et al., assesses the impact of chronic cough on quality of life across physical, psychological, and social domains. Total scores range from 3 to 21, with higher scores indicating less impairment [27].

The SSD-12 assesses cognitive, emotional, and behavioral responses to somatic symptoms. The scale contains 12 items with total scores ranging from 0 to 48; scores ≥ 16 indicate significant somatic symptom burden [28].

The PHQ-15 evaluates the severity of common somatic symptoms (e.g., gastrointestinal discomfort, headache, chest tightness, fatigue) over the past 4 weeks. Total scores range from 0 to 30, with ≥ 15 indicating a high somatic symptom burden [29].

The GAD-7 assesses anxiety severity over the past 2 weeks, with total scores ranging from 0 to 21; scores ≥ 5 indicate clinically relevant symptoms [30].

The PHQ-9 evaluates depressive symptoms over the past 2 weeks, with total scores ranging from 0 to 27; scores ≥ 5 suggest abnormality [31].

Capsaicin cough sensitivity testing was conducted using a modified method based on Fujimura et al. The lowest concentrations of capsaicin required to elicit two coughs (C2) and five coughs (C5) were recorded; lower threshold values indicate greater cough sensitivity [32].

### 2.2. Statistical Analysis

All statistical analyses were performed using SPSS version 26.0 (IBM Corp., Armonk, NY, USA) and R version 4.3.2 (R Foundation for Statistical Computing, Vienna, Austria). The normality of continuous variables was assessed using the Shapiro–Wilk test. Normally distributed data are presented as mean ± standard deviation (SD) and compared using independent-samples t-test. Non-normally distributed data are expressed as median and interquartile range (IQR) and compared using the Mann–Whitney U test. Categorical variables are presented as frequencies and percentages and compared using the χ^2^ test.

Correlations between continuous variables were evaluated using Spearman correlation analysis according to data distribution. Variables with *p* < 0.05 in univariate analyses were entered into a binary logistic regression model to identify independent factors for SSD in patients with GERC. Multivariable logistic regression was performed using a forward stepwise method (Forward: LR). In addition, Firth’s penalized logistic regression was performed as a sensitivity analysis. Prior to multivariate modeling, collinearity diagnostics were conducted using variance inflation factors (VIF), with VIF > 5 indicating multicollinearity; corresponding variables were excluded. Model fit was assessed using Nagelkerke R^2^, and classification accuracy was used as an indicator of model performance.

All statistical tests were two-tailed, and *p* < 0.05 was considered statistically significant. Odds ratios (ORs) and 95% confidence intervals (CIs) for significant variables were visualized using forest plots generated with the “ggplot2” package in R.

## 3. Results

### 3.1. Demographic Characteristics

A total of 249 patients with GERC were initially screened, of whom 215 completed both the questionnaires and the structured interview; their data were included in the final analysis. The patient enrollment flow is illustrated in Figure 1. The mean age of the patients was 51.1 years (SD 14.7), with 82 (38.1%) males and 133 (61.9%) females. The median cough duration was 24 months, and the median number of healthcare visits was 4. Among the participants, 106 (49.3%) showed symptom improvement following standard anti-reflux therapy, whereas 109 (50.7%) required intensified anti-reflux treatment to achieve symptom relief. The clinical characteristics and results of cough- and psychological assessments are summarized in Table 1.

### 3.2. Prevalence of SSD in Patients with GERC

**Figure 2:**
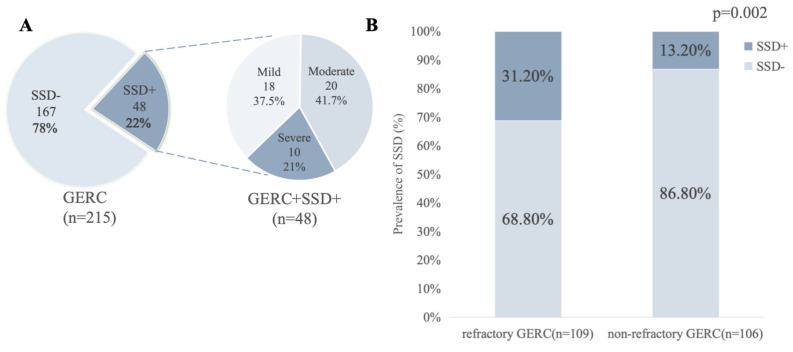
Prevalence and severity distribution of SSD in patients with GERC. (**A**) The left panel shows the proportion of patients with and without SSD in the overall GERC cohort (n = 215). The right panel illustrates the distribution of mild, moderate, and severe SSD among SSD-positive patients only (n = 48), with percentages calculated within the SSD-positive subgroup. (**B**) Comparison of SSD prevalence between refractory and non-refractory GERC groups.

Among the 215 patients with GERC, 101 (47.0%) met the screening criteria and were referred for MDT evaluation, while 114 did not undergo SCID assessment. Among those referred, 48 (47.5%) were finally diagnosed with SSD. The detailed stratification of screening patterns and corresponding diagnostic outcomes is provided in Supplementary Table S1. Mild, moderate, and severe SSD accounted for 8.4% (18/215), 9.3% (20/215), and 4.7% (10/215) of cases, respectively (Figure 2A). Among patients with refractory GERC, the prevalence of SSD was 31.2% (34/109), significantly higher than that in patients who responded to standard anti-reflux therapy (13.2%, 14/106) (OR = 2.98, 95% CI: 1.49–5.95, *p* = 0.002) (Figure 2B). To further evaluate the robustness of this association, stratified analyses based on key clinical variables were conducted, showing a consistent direction of association across all subgroups; detailed results are presented in Figure S1. Based on questionnaire assessments, 34.0% (73/215) of GERC patients had anxiety (GAD-7 ≥ 5), and 35.4% (76/215) had depressive symptoms (PHQ-9 ≥ 5). Among patients with SSD, 35 (72.9%) exhibited positive scores for anxiety or depression.

### 3.3. Clinical and Psychosomatic Features in GERC Patients with and Without SSD

Based on SSD diagnosis established by structured clinical interview, patients with GERC were classified into the SSD+ and SSD− groups. The two groups were compared with respect to baseline clinical characteristics, MII-pH findings, and cough- and psychosomatic-related questionnaire scores.

The SSD+ group had a significantly higher healthcare visit (median: 8 vs. 4). Regarding cough characteristics, C2, C5, daytime cough symptom scores, and HARQ scores were all significantly higher in the SSD+ group than in the SSD− group (*p* < 0.05). In addition, the SSD+ group showed lower LCQ scores and higher PHQ-15, GAD-7, and PHQ-9 scores (all *p* < 0.05), indicating that patients with SSD exhibited not only more pronounced cough hypersensitivity but also greater somatization, anxiety, and depressive symptoms (Table 2). Further analysis revealed no significant differences between the two groups in MII-pH parameters (Table 3 and Supplementary Table S2). However, the SSD+ group demonstrated a markedly poorer response to standard anti-reflux therapy (*p* < 0.05), suggesting that SSD may contribute to symptom persistence and reduced treatment responsiveness in patients with GERC. 

### 3.4. Correlation Between SSD and HARQ Scores in Patients with GERC

**Figure 3:**
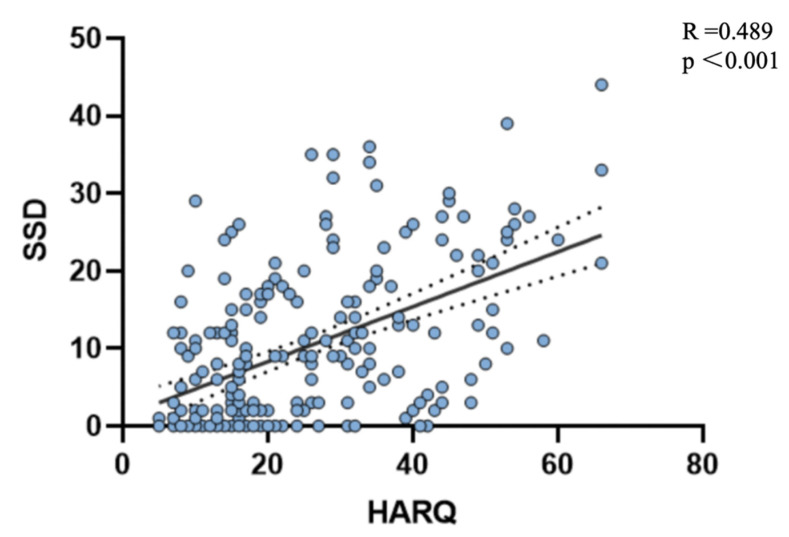
Correlation between HARQ score and SSD-12 score in patients with GERC.A moderate positive correlation was observed (R = 0.489, *p* < 0.001).

The HARQ is commonly used to assess airway reflux and cough hypersensitivity. Spearman correlation analysis revealed a significant positive correlation between HARQ and SSD-12 scores (r = 0.489, *p* < 0.05) (Figure 3). In addition, comparison of HARQ item scores between the SSD+ and SSD− groups showed that patients in the SSD+ group scored significantly higher on most items, including “itchy throat or globus sensation,” “coughing while eating,” “cough triggered by specific foods,” and “cough provoked by singing,” among others (*p* < 0.05). These findings suggest that patients with GERC and SSD exhibit heightened cough reflex sensitivity. Detailed results are presented in Supplementary Table S3.

### 3.5. Analysis of Associated Factors for SSD in Patients with GERC

**Figure 4:**
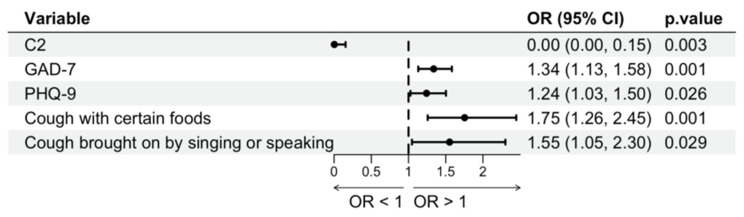
Forest plot showing independent correlate factors associated with SSD in patients with GERC. Variables including cough sensitivity (C2), anxiety (GAD-7), depression (PHQ-9), and cough triggers are presented as odds ratios (ORs) with 95% confidence intervals (CIs). The dashed vertical line represents OR = 1.

To further identify factors associated with SSD in patients with GERC, variables that showed differences between the SSD+ and SSD− groups, along with clinically relevant variables, were included in a binary logistic regression analysis. Univariate analysis revealed that the number of healthcare visits, C2, C5, daytime CSS, HARQ score, LCQ score, PHQ-15, GAD-7, and PHQ-9 scores were all significantly associated with SSD (*p* < 0.05) (Supplementary Table S4). These variables were subsequently entered into a multivariable stepwise logistic regression model (Forward: LR) to construct a prediction model (Table 4). The results showed that C2, GAD-7 score, PHQ-9 score, “cough triggered by specific foods,” and “cough induced by talking or singing” were independent correlate factors for SSD in patients with GERC (*p* < 0.05). The model demonstrated good model fit (Nagelkerke R^2^ = 0.738). The overall classification accuracy was 89.8%, with a correct identification rate of 94.6% for non-SSD patients and 72.9% for SSD patients, indicating strong predictive performance. To further assess the robustness of the results, Firth’s penalized logistic regression was performed as a sensitivity analysis, which yielded consistent findings (see Supplementary Table S5). A forest plot illustrating the ORs and 95% confidence intervals for these variables is presented in Figure 4.

## 4. Discussion

To our knowledge, this study is the first to systematically evaluate the prevalence and clinical characteristics of SSD in patients with GERC. Our results showed that the prevalence of SSD in this population was 22.3%. Increased capsaicin cough sensitivity, cough triggered by specific foods, cough induced by talking or singing, and higher levels of anxiety and depression were identified as independent factors for SSD in patients with GERC.

Previous studies have primarily focused on the impact of emotional disorders—such as anxiety and depression—on reflux symptoms and disease course in patients with GERD [10]. However, research on SSD in patients with GERC remains scarce. In the present study, the prevalence of SSD in patients with GERC (22.3%) was lower than that reported in general outpatient populations (33.8%) [17], but higher than the 10–15% reported in the general population [33].

In this study, patients in the SSD+ group exhibited more frequent healthcare utilization and poorer cough-related quality of life, which may be related to heightened bodily attention and catastrophic interpretation of symptoms. Individuals with SSD are more sensitive to minor physical discomfort and may engage in repeated healthcare-seeking behaviors. This pattern of increased healthcare utilization has also been documented in other chronic psychosomatic conditions, important role of cognitive and emotional processes in shaping illness behavior [12,14].

Patients with GERC combined with SSD show a poorer response to standard anti-reflux treatment and demonstrate heightened cough sensitivity, suggesting that SSD may be associated with the symptom experience and treatment response of GERC through mechanisms involving symptom amplification and central neural processing. On one hand, SSD patients tend to magnify and catastrophize mild discomfort, resulting in a reduced perception of symptom improvement [11]. On the other hand, In the present study that SSD+ patients exhibited higher cough sensitivity. Previous research has shown that patients with increased cough sensitivity exhibit elevated neural activity in the periaqueductal gray matter, an area involved in the emotional processing of stress and pain [34,35]. Our earlier work has demonstrated that antidepressants, such as flupentixol-melitracen, can alleviate cough symptoms in RCC patients by reducing the heightened sensitivity of the cough center [36]. These findings support the hypothesis that the central emotional processing abnormalities in SSD patients may overlap with mechanisms that increase cough sensitivity, collectively contributing to the persistence of subjective symptoms and incomplete treatment responses.

This study found that elevated levels of anxiety and depression are independent correlate factors for SSD in patients with GERC, consistent with previous findings [37]. Emotional disorders are closely associated with the onset and persistence of SSD. Individuals with anxiety and depression tend to exhibit increased attentional focus on bodily sensations and catastrophize them, which may contribute to symptom amplification and somatization. Furthermore, anxiety and depression are common in GERD and chronic cough, where they can influence reflux and cough perception by altering autonomic nervous activity and visceral sensation thresholds, potentially contributing to the development of SSD [38,39,40]. These findings suggest that negative emotions may play an important role in the biopsychosocial interactions underlying GERC.

This study is the first to explore the prevalence and clinical characteristics of SSD in patients with GERC, but it also has several limitations. First, the sample size of this study was relatively small, and the single-center design may have introduced selection bias, thereby limiting the generalizability of the findings. In addition, the relatively small number of SSD-positive cases may introduce small-sample bias and potential separation issues in logistic regression analyses, which may affect the stability of parameter estimates and the precision of the results. Second, this study adopted a screening-based diagnostic approach, in which SCID were performed only in patients who screened positive on psychological questionnaires, while those with negative screening results did not undergo further evaluation, which may have led to an underestimation of the true prevalence of SSD. Third, in this study, the association between SSD and response to anti-reflux therapy was based on correlation analyses and therefore does not imply causality or predictive value; potential confounding factors also cannot be fully excluded. Future studies should construct multivariable models with treatment response as the outcome and incorporate a broader range of potential influencing factors to more comprehensively evaluate the independent role of SSD in treatment response. Fourth, this study did not further investigate the central functional changes in GERC patients with SSD using techniques such as functional magnetic resonance imaging.

Based on the findings of the present study, future research should further explore comprehensive intervention strategies for patients with GERC coexisting with SSD. In terms of pharmacological interventions, serotonin-norepinephrine reuptake inhibitors (SNRIs) have been recommended by clinical guidelines for the treatment of SSD [41]. Our previous randomized controlled trial further demonstrated that duloxetine, an SNRI, significantly improved cough symptoms in patients with RCC, suggesting that it may reduce cough hypersensitivity through central mechanisms [42]. Based on these findings, future studies could be designed to investigate the effects of combining SNRIs with anti-reflux therapy in patients with GERC and SSD, and to further evaluate the potential underlying mechanisms. In addition, non-pharmacological interventions warrant further attention. Psychological and behavioral therapies targeting SSD, such as cognitive-behavioral therapy (CBT) or mindfulness-based interventions, may improve overall symptom burden by modulating symptom perception and emotional responses. Future studies may explore whether combining these approaches with anti-reflux therapy can further improve cough symptoms and quality of life in patients with GERC and SSD.

Overall, future research should expand sample sizes, adopt multicenter study designs, and incorporate neuroimaging approaches to further elucidate the interaction mechanisms between SSD and GERC, as well as to evaluate the clinical efficacy of anti-reflux therapy combined with pharmacological and non-pharmacological interventions targeting SSD in improving cough symptoms and overall symptom burden.

## 5. Conclusions

The prevalence of SSD in patients with GERC is 22.3%. Cough hypersensitivity, as well as psychiatric disorders such as anxiety and depression, are independent correlate factors for GERC patients with SSD. The findings of this study suggest that emotional disorders and biopsychosocial comorbidities should be given greater attention in the clinical management of GERC patients. For patients who exhibit poor response to standard anti-reflux therapy or persistent symptoms, early identification of psychiatric disorders such as SSD, along with the integration of biopsychosocial treatments, may help improve treatment outcomes.

## Figures and Tables

**Table 1:** Baseline demographic and clinical characteristics of patients with GERC.

	GERC (*n* = 215)
Age(y)	51.09 ± 14.74
Gender (M/F)	82/133
BMI (kg/m^2^)	24.27 ± 4.26
Duration (m)	24.00 (54.00)
Doctor visit times	4.00 (4.00)
Lung function	
FEV1 predicted (%)	99.25 ± 13.69
FVC predicted (%)	97.79 ± 13.37
FEV1/FVC% max	100.00 ± 7.98
Capsaicin cough sensitivity	
C2	0.95 ± 0.39
C5	1.16 ± 0.48
Cough symptom score	
Daytime	3.00 (2.00)
Nighttime	1.00 (1.00)
HARQ	22.00 (19.00)
GerdQ	8.00 (4.00)
LCQ score	12.64 (3.83)
SSD-12 score	8.00 (15.00)
PHQ-15 score	4.00 (9.00)
GAD-7 score	2.00 (7.00)
PHQ-9 score	3.00 (6.00)
The effect of standard anti-reflux(Y/N)	106/109

Note: Data are presented as mean ± standard deviation, median (interquartile range), or No. (%) unless otherwise indicated. GERC, gastroesophageal reflux related chronic cough; BMI, body mass index; FEV1, forced expiratory volume in 1 s; FVC, forced vital capacity; C2, capsaicin solution concentration with ≥2 coughs; C5, capsaicin solution concentration with ≥5 coughs; HARQ, Hull airway reflux questionnaire; GerdQ, gastroesophageal reflux disease questionnaire; LCQ, Leicester cough questionnaire; SSD-12, somatic symptom disorder-B criteria scale; PHQ-15, patient health questionnaire-15; GAD-7, general anxiety disorder-7; PHQ-9, patient health questionnaire-9.

**Table 2:** Comparison of baseline demographic and clinical characteristics between SSD+ and SSD− patients with GERC.

	SSD− (*n* = 167)	SSD+ (*n* = 48)	Test Results
Age(y)	51.65 ± 14.49	49.15 ± 15.56	t = 1.039, *p* = 0.300
Gender (M/F)	58/109	24/24	χ^2^ = 3.684, *p* = 0.055
BMI (kg/m^2^)	24.62 ± 4.53	23.92 ± 3.12	t = 1.004, *p* = 0.317
Duration (m)	24.00 (54.00)	24.00 (54.00)	Z = −0.295, *p* = 0.768
Doctor visit times	4.00 (3.00)	8.00 (7.00) **	Z = −5.316, *p* < 0.001
Lung function			
FEV1 predicted (%)	99.03 ± 13.69	99.97 ± 13.80	t = −0.418, *p* = 0.677
FVC predicted (%)	97.51 ± 13.34	99.56 ± 13.49	t = −0.935, *p* = 0.351
FEV1/FVC% max	100.14 ± 8.10	99.63 ± 7.62	t = 0.389, *p* = 0.689
Capsaicin cough sensitivity			
C2	1.02 ± 0.42	0.74 ± 0.13 **	t = 7.319, *p* < 0.001
C5	1.21 ± 0.50	0.98 ± 0.37 *	t = 3.541, *p* = 0.001
Cough symptom score			
Daytime	3.00 (2.00)	3.00 (1.00) *	Z = −2.175, *p* = 0.030
Nighttime	1.00 (1.00)	2.00 (2.00)	Z = −1.175, *p* = 0.240
HARQ	19.00 (16.00)	34.50 (26.00) **	Z = −5.511, *p* < 0.001
GerdQ	7.00 (4.00)	8.00 (3.00)	Z = −1.608, *p* = 0.108
LCQ score	13.27 (3.36)	10.55 (5.48) **	Z = −5.571, *p* < 0.001
PHQ-15 score	2.00 (7.00)	11.00 (7.00) **	Z = −7.248, *p* < 0.001
GAD-7 score	0.00 (3.00)	7.50 (7.00) **	Z = −8.997, *p* < 0.001
PHQ-9 score	1.00 (15.00)	8.00 (6.00) **	Z = −8.387, *p* < 0.001
The effect of standard anti-reflux (Y/N)	92/75	14/34 *	χ^2^ = 10.024, *p* = 0.002

Note: Data are presented as mean ± standard deviation, median (interquartile range), or No. (%) unless otherwise indicated. GERC, gastroesophageal reflux related chronic cough; SSD, somatic symptom disorder; BMI, body mass index; FEV1, forced expiratory volume in 1 s; FVC, forced vital capacity; C2, capsaicin solution concentration with ≥2 coughs; C5, capsaicin solution concentration with ≥5 coughs; HARQ, Hull airway reflux questionnaire; GerdQ, gastroesophageal reflux disease questionnaire; LCQ, Leicester cough questionnaire; SSD-12, somatic symptom disorder-B criteria scale; PHQ-15, patient health questionnaire-15; GAD-7, general anxiety disorder-7; PHQ-9, patient health questionnaire-9.* Compared with SSD− group, *p* < 0.05; ** compared with SSD− group, *p* < 0.001.

**Table 3:** Comparison of MII-pH monitoring results between SSD+ and SSD− patients with GERC.

	SSD− (*n* = 167)	SSD+ (*n* = 48)	Test Results
AET (%)	0.70 (2.70)	1.15 (2.90)	Z = −0.603, *p* = 0.547
DeMeester score	2.98 (7.93)	3.60 (12.95)	Z = −0.671, *p* = 0.502
Total reflux (n)	100.00 (90.00)	127.00 (107.00)	Z = −1.642, *p* = 0.101
Acidic reflux (n)	10.50 (24.00)	13.50 (20.00)	Z = −0.660, *p* = 0.509
Weakly acidic reflux (n)	49.00 (50.00)	67.00 (51.00)	Z = −1.137, *p* = 0.255
Weakly alkaline reflux (n)	24.00 (43.00)	35.50 (77.00)	Z = −0.921, *p* = 0.357
Type of refluxate			
Gas reflux (n)	25.00 (26.00)	31.00 (30.00)	Z = −1.369, *p* = 0.171
Liquid reflux (n)	26.00 (34.00)	35.50 (53.00)	Z = −1.006, *p* = 0.314
Mixed reflux (n)	33.00 (40.00)	41.00 (65.00)	Z = −1.345, *p* = 0.179
SAP (%)	85.20 (30.20)	85.90 (33.30)	Z = −0.061, *p* = 0.951
Acid SAP (%)	0.00 (94.50)	0.00 (95.60)	Z = −0.507, *p* = 0.612
Nonacid SAP (%)	95.70 (9.60)	95.00 (18.02)	Z = 0.727, *p* = 0.467
SI (%)	33.30 (25.00)	33.30 (27.60)	Z = −0.030, *p* = 0.976

Note: Data are presented as median (interquartile range), or No. (%) unless otherwise indicated. MII-pH, multichannel intraluminal impedance-pH monitoring; SSD, somatic symptom disorder; GERC, gastroesophageal reflux related chronic cough; AET, acid exposure time; SAP, symptom association probability; SI, symptom index.

**Table 4:** Multivariate stepwise logistic regression identifying independent correlate factors for SSD in patients with GERC.

Variable	*B*	Wald	*p* Value	OR	95%CI
C2	−5.674	8.542	0.003	0.003	0.000–0.154
GAD-7	0.291	18.514	0.001	1.338	1.131–1.583
PHQ-9	0.217	4.964	0.026	1.242	1.026–1.503
Cough with certain foods	0.562	10.906	0.001	1.754	1.257–2.448
Cough brought on by singing or speaking	0.440	4.778	0.029	1.552	1.046–2.302

Note: Stepwise logistic regression (Forward: LR). OR = odds ratio; CI = confidence interval. Nagelkerke R^2^ = 0.738; C2, capsaicin solution concentration with ≥2 coughs; GAD-7, general anxiety disorder-7; PHQ-9, patient health questionnaire-9.

## Data Availability

The data that support the findings of this study are available from the corresponding author upon reasonable request.

## Supplementary Materials

The following supporting information can be downloaded at: https://www.mdpi.com/article/10.3390/jcm15103618/s1, Supplementary Material S1. Supplementary Table S1. Screening patterns and final SSD diagnosis among patients referred for MDT evaluation; Supplementary Table S2. Distribution of acidic and non-acidic GERC and diagnostic patterns; Supplementary Table S3. Comparison of individual HARQ item scores between SSD+ and SSD− patients with GERC; Supplementary Table S4. Univariate logistic regression analysis of risk factors for SSD in patients with GERC; Supplementary Table S5. Multivariable Firth penalized logistic regression identifying independent factors associated with SSD in patients with GERC; Figure S1. Stratified analysis of the association between somatic symptom disorder (SSD) and poor response to standard anti-reflux therapy in patients with GERC. Supplementary Material S2. Structured Clinical Interview for DSM-5 (SCID) used for psychiatric assessment.

## Abbreviations

The following abbreviations are used in this manuscript:

GERC	Gastroesophageal reflux-induced chronic cough
GERD	Gastroesophageal reflux disease
SSD	Somatic symptom disorder
DSM-5	Diagnostic and Statistical Manual of Mental Disorders, Fifth Edition
BMI	Body mass index
CSS	Cough Symptom Score
HARQ	Hull Airway Reflux Questionnaire
GerdQ	Gastroesophageal Reflux Disease Questionnaire
LCQ	Leicester Cough Questionnaire
PHQ-15	Patient Health Questionnaire-15
SSD-12	Somatic Symptom Disorder-12
PHQ-9	Patient Health Questionnaire-9
GAD-7	Generalized Anxiety Disorder-7
MII-pH	Multichannel intraluminal impedance-pH
PPI	Proton pump inhibitor
AET	Acid exposure time
SAP	Symptom association probability
SI	Symptom index
PPIs	Proton pump inhibitors
MDT	Multidisciplinary team
SCID	Structured Clinical Interview
LES	Lower esophageal sphincter
C2	The lowest concentration of inhaled capsaicin that provokes ≥ 2 coughs
C5	The lowest concentration of inhaled capsaicin that provokes ≥ 5 coughs
SNRIs	Serotonin-norepinephrine reuptake inhibitors
CBT	Cognitive-behavioral therapy
