# Supplementary material for: Somatic Symptom Disorder Is Associated with Cough Hypersensitivity and Poor Response to Anti-Reflux Therapy in Patients with Gastroesophageal Reflux-Induced Chronic Cough
Source: J Clin Med. 2026 May 8;15(10):3618. doi: 10.3390/jcm15103618 (PMC13207722; doi:10.3390/jcm15103618)
Supplement: Supplementary file 1 [file jcm-15-03618-s001.zip › Supplementary Material_S1.pdf]

Prevalence and characteristics of somatic symptom disorder in patients with gastroesophageal reflux-induced chronic cough
Supplementary file

Supplementary Table S1. Screening patterns and final SSD diagnosis among patients referred for MDT evaluation

Screening pattern	N to MDT	SSD+ (n)	SSD− (n)	SSD+ (%)
SSD12+ & PHQ9+ & GAD7+	40	29	11	72.5
PHQ9+ & GAD7+	17	3	14	17.6
PHQ9+ only	15	1	14	6.7
GAD7+ only	9	2	7	22.2
SSD12+ only	9	4	5	44.4
SSD12+ & GAD7+	7	5	2	71.4
SSD12+ & PHQ9+	4	4	0	100
Total (referred)	101	48	53	47.5

Note: MDT, multidisciplinary team; SSD, somatic symptom disorder; SSD-12, Somatic Symptom Disorder－B Criteria Scale (positive⩾16); PHQ-9, Patient Health Questionnaire-9 (positive⩾5); GAD-7, Generalized Anxiety Disorder-7 (positive⩾5).

Supplementary Table S2. Distribution of acidic and non-acidic GERC and diagnostic patterns

Category	n (%)
Acid GERC	87 (40.5%)
Acid SAP positive only	52 (59.8%)
AET positive only	26 (29.9%)
Both AET and acid SAP positive	9 (10.3%)
Non-acid GERC	128 (59.5%)
Total GERC	215 (100.0%)

Note: GERC, gastroesophageal reflux–induced chronic cough; AET, acid exposure time; SAP, symptom association probability; Acid SAP positivity and non-acid SAP positivity were defined as SAP ⩾ 95%; Abnormal acid exposure was defined as AET⩾6%.

Supplementary Table S3. Comparison of individual HARQ item scores between SSD+ and SSD− patients with GERC

Item	HARQ	SSD- (n=167)	SSD+ (n=48)	Test results
1	Hoarseness or a problem with your voice	1.21±1.35	1.33±1.39	t=-0.606, P=0.544
2	Clearing your throat	1.80±1.65	3.44±1.22**	t=-5.930,P＜0.001
3	Excess mucus in the throat, or drip down the back of your nose	1.78±1.61	2.10±1.59	t=-1.288, P=0.198
4	Retching or vomiting when you cough	1.49±1.52	1.71±1.44	t=-1.146, P=0.252
5	Cough on first lying down or bending over	1.81±1.49	2.73±1.58**	t=-3.628, P＜0.001
6	Chest tightness or wheeze when coughing	1.34±1.62	1.54±1.56	t=-1.058, P=0.290
7	Heartburn, indigestion, stomach acid coming up (or do you take medications for this, if yes score 5)	2.16±1.66	2.52±1.74	t=-1.260, P=0.208
8	A tickle in your throat, or a lump in your throat	0.96±1.28	3.04±1.60**	t=-7.326, P＜0.001
9	Cough with eating (during or soon after meals)	0.98±1.54	3.08±1.69**	t=-7.028, P＜0.001
10	Cough with certain foods	2.04±1.46	3.98±1.25**	t=-7.181, P＜0.001
11	Cough when you get out of bed in the morning	1.61±1.62	2.25±1.78*	t=-2.228, P=0.026
12	Cough brought on by singing or speaking (for example, on the telephone)	2.18±1.74	3.29±1.37**	t=-3.960, P＜0.001
13	Coughing during the day rather than night	2.04±1.59	3.25±1.64**	t=-4.323, P＜0.001
14	A strange taste in your mouth	1.08±1.40	1.13±1.25	t=-0.541, P=0.588

Note: Data are presented as mean ± standard deviation. HARQ, Hull airway reflux questionnaire; GERC, gastroesophageal reflux related chronic cough; SSD, somatic symptom disorder. * Compared with SSD－ group, P<0.05; ** compared with SSD－ group, P<0.001.

Supplementary Table S4. Univariate logistic regression analysis of risk factors for SSD in patients with GERC

	OR	95%CI	p value
C2	0.010	0.001-0.099	＜0.001
C5	0.269	0.110-0.659	0.004
Daytime CSS	1.475	1.005-2.163	0.047
LCQ score	0.711	0.629-0.804	＜0.001
PHQ-15 score	1.293	1.195-1.398	＜0.001
GAD-7 score	1.524	1.347-1.723	＜0.001
PHQ-9 score	1.499	1.327-1.693	＜0.001
Clearing your throat	1.951	1.527-2.491	＜0.001
Cough on first lying down or bending over	1.486	1.189-1.856	＜0.001
A tickle in your throat, or a lump in your throat	2.349	1.832-3.013	＜0.001
Cough with eating (during or soon after meals)	1.908	1.563-2.329	＜0.001
Cough with certain foods	2.625	1.945-3.543	＜0.001
Cough when you get out of bed in the morning	1.251	1.035-1.512	0.021
Cough brought on by singing or speaking (for example, on the telephone)	1.518	1.223-1.884	＜0.001
Coughing during the day rather than night	1.599	1.284-1.991	＜0.001

Note: Univariate logistic regression analysis was performed to identify potential risk factors for SSD in patients with GERC. OR = odds ratio; CI = confidence interval. C2, capsaicin solution concentration with ≥2 coughs; C5, capsaicin solution concentration with ≥5 coughs; CSS, cough symptom score; LCQ, Leicester cough questionnaire; SSD-12, somatic symptom disorder-B criteria scale; PHQ-15, patient health questionnaire-15; GAD-7, general anxiety disorder-7; PHQ-9, patient health questionnaire-9.

Supplementary Table S5. Multivariable Firth penalized logistic regression identifying independent factors associated with SSD in patients with GERC

Variable	B	p value	OR	95%CI
C2	-2.102	<0.001	0.112	0.025-0.392
GAD-7	0.256	<0.001	1.292	1.126-1.511
PHQ-9	0.192	0.019	1.212	1.032-1.443
Cough with certain foods	0.184	0.250	1.202	0.878-1.660
Cough brought on by singing or speaking	0.417	0.021	1.518	1.064-2.261

Note: Firth penalized logistic regression was performed as a sensitivity analysis to address potential small-sample bias and quasi-complete separation. OR = odds ratio; CI = confidence interval. Nagelkerke R^2=0.738； C2, capsaicin solution concentration with ≥2 coughs; GAD-7, general anxiety disorder-7; PHQ-9, patient health questionnaire-9.

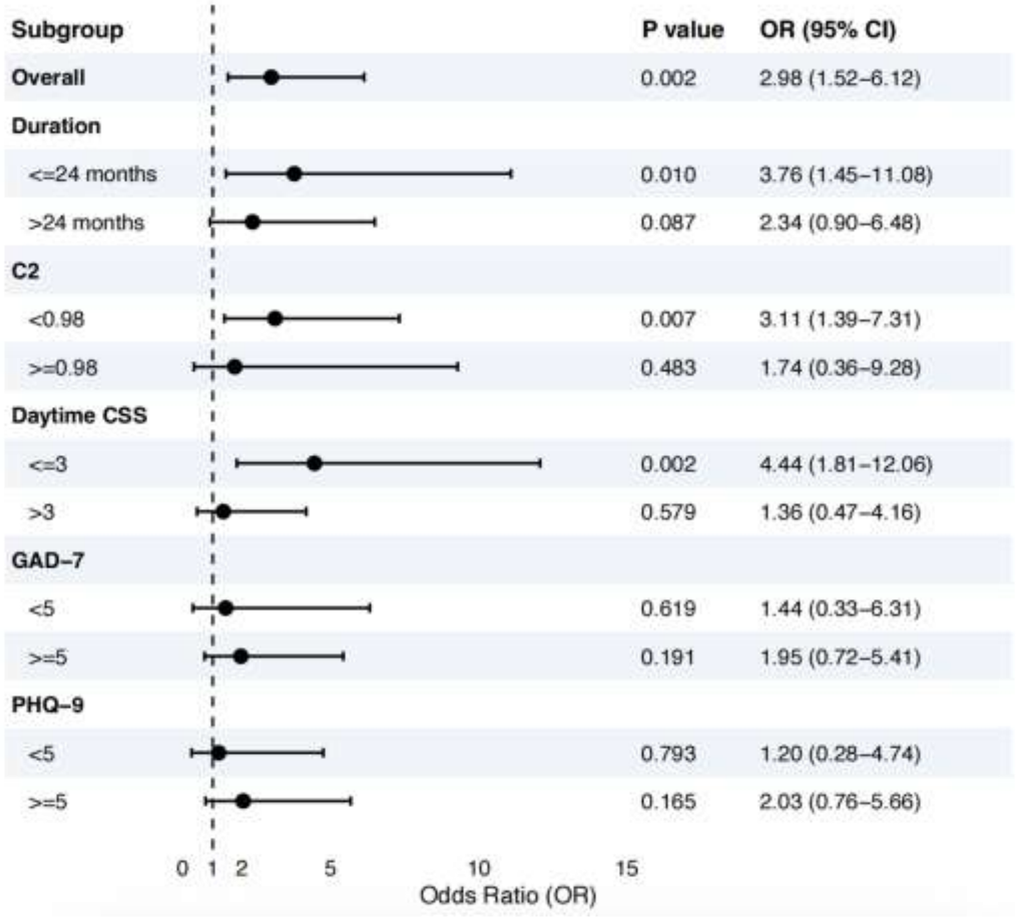

Figure S1. Stratified analysis of the association between somatic symptom disorder (SSD) and poor response to standard anti-reflux therapy in patients with GERC.

Forest plot showing the association between somatic symptom disorder (SSD) and poor response to standard anti-reflux therapy across predefined subgroups. Stratification was performed according to cough duration (≤24 vs. >24 months), cough sensitivity (C2 <0.98 vs. ≥0.98), daytime cough symptom score (CSS ≤3 vs. >3), anxiety status (GAD-7 <5 vs. ≥5), and depressive symptoms (PHQ-9 <5 vs. ≥5). Odds ratios (ORs) with 95% confidence

intervals (CIs) are presented for each subgroup. The dashed vertical line indicates an OR of 1. Across all subgroups, the direction of association remained consistent, supporting the robustness of the main findings.
