# Supplementary material for: Somatic Symptom Disorder Is Associated with Cough Hypersensitivity and Poor Response to Anti-Reflux Therapy in Patients with Gastroesophageal Reflux-Induced Chronic Cough
Source: J Clin Med. 2026 May 8;15(10):3618. doi: 10.3390/jcm15103618 (PMC13207722; doi:10.3390/jcm15103618)
Supplement: Supplementary file 1 [file jcm-15-03618-s001.zip › Supplementary Material_S2.pdf]

J. SOMATIC SYMPTOM AND RELATED DISORDERS (OPTIONAL)

SOMATIC SYMPTOM DISORDER (OPTIONAL) (CURRENT ONLY)	SOMATIC SYMPTOM DISORDER CRITERIA					
IF SCREENING QUESTION #13d ANSWERED "NO," SKIP TO ***ILLNESS ANXIETY DISORDER* Opt-***J.3.* <br>*IF SCREENING QUESTION #13d ANSWERED "YES":* **You've said that over the past 6 months, since** (6 MONTHS AGO), **you have been bothered by physical symptoms.**		SCREEN Q#11d<br>YES | | NO<br><br>IF NO, GO TO ***ILLNESS ANXIETY DISORDER*** Opt-J.3				OJ1
IF SCREENER NOT USED: **Over the past 6 months, since** (6 MONTHS AGO)**, have you had any physical symptoms that were distressing to you or that affected your day-to-day life?** <br>**Tell me about that. (How much have** (SYMPTOM[S]) **bothered you? How much have** (SYMPTOM[S]) **interfered with your daily life? Have you changed your day-to-day activities in any way?)**	A. One or more somatic symptoms that are distressing or result in significant disruption of daily life.	?	1<br><br>GO TO ***ILLNESS ANXIETY DISORDER*** Opt-J.3	2	3	OJ2
	B. Excessive thoughts, feelings, or behaviors related to the somatic symptoms or associated health concerns as manifested by at least one of the following:					
**How concerned are you about your symptoms? What are you concerned about? (Have you gone to the doctor about this? What did he or she say? What did you think? Are you more concerned than the doctor suggests you need to be?)** <br>**Do other people (like family or friends) think you worry too much about** (SYMPTOM[S])**?** <br>**How much do you think about this?** <br>**Do you have trouble thinking about other things in your life because of these concerns?**	1. Disproportionate and persistent thoughts about the seriousness of one's symptoms.	?	1	2	3	OJ3
**How anxious are you about your overall health? Do friends, family, or your doctors think you worry too much about your health?** <br>*IF NO:* **Do you get very anxious whenever you notice a physical symptom? (Tell me about that.)** <br>**How long does this anxiety last?**	2. Persistently high level of anxiety about health or symptoms.	?	1	2	3	OJ4

?=inadequate information 1=absent or false 2=subthreshold 3=threshold or true

Interview question	Criterion	Rating	Code
**Over the past 6 months, since** (6 MONTHS AGO), **how much time and energy have you spent...**<br><br>**...thinking about** (SXS) **or your health?**<br><br>**...going to doctors or getting tests done?**<br><br>**...looking up your symptoms on the internet or in books?**<br><br>**....shopping for supplements or treatments in stores or on the internet?**<br><br>**...talking to friends, family members, or co-workers about your symptoms or your health?**<br><br>**(How often do you check your body for signs of illness, like looking at your throat in the mirror or checking your body for lumps?)**	3. Excessive time and energy devoted to these symptoms or health concerns.	? 1 2 3	OJ5
	AT LEAST ONE "B" SYMPTOM IS CODED "3"	? 1 3<br>1: GO TO ***ILLNESS ANXIETY DISORDER*** Opt-J.3	OJ6
IF UNCLEAR: **For most of the time during the past 6 months, have you had physical symptoms of one kind or another?**	C. Although any one somatic symptom may not be continuously present, the state of being symptomatic is persistent (typically more than 6 months).	? 1 2 3<br>1: GO TO ***ILLNESS ANXIETY DISORDER*** Opt-J.3	OJ7
	CRITERIA A, B, AND C ARE CODED "3"	1 3<br>1: GO TO ***ILLNESS ANXIETY DISORDER*** Opt-J.3<br>3: SOMATIC SYMPTOM DISORDER	OJ8
IF UNKNOWN: **How old were you when you first started being very concerned about your health or physical symptoms?**	Age-at-onset of Somatic Symptom Disorder (CODE 99 IF UNKNOWN)	____ ____	OJ9
IF UNKNOWN: **Of all of these symptoms, which bothers you the most?**	*Specify if:* (check all that apply)		
	___ **With predominant pain**: if somatic symptoms predominantly involve pain		OJ10
	___ **Persistent:** if course is characterized by severe symptoms, marked impairment, and long duration (more than 6 months)		OJ11
	Specify ***severity****:* (circle the appropriate number)<br>1 - **Mild:** Only one of the symptoms specified in Criterion B are fulfilled<br>2 - **Moderate:** Two or more of the symptoms specified in Criterion B are fulfilled.<br>3 - **Severe:** Two or more of the symptoms specified in Criterion B are fulfilled, plus there are multiple somatic complaints (or one very severe somatic symptom).		OJ12
